# Associations of congenital heart disease with deprivation index by rural-urban maternal residence: a population-based retrospective cohort study in Ontario, Canada

**DOI:** 10.1186/s12887-022-03498-6

**Published:** 2022-08-05

**Authors:** Qun Miao, Sandra Dunn, Shi Wu Wen, Jane Lougheed, Fayza Sharif, Mark Walker

**Affiliations:** 1 BORN Ontario, Children’s Hospital of Eastern Ontario, Ottawa, Ontario Canada; 2 Children’s Hospital of Eastern Ontario Research Institute, Ottawa, Ontario Canada; 3 School of Epidemiology and Public Health, University of Ottawa Faculty of Medicine, Ottawa, Canada; 4 OMNI Research Group, Clinical Epidemiology Program, Ottawa Hospital Research Institute, Ottawa, Canada; 5 School of Nursing, University of Ottawa, Ottawa, Ontario Canada; 6 Department of Obstetrics & Gynecology, University of Ottawa, Faculty of Medicine, Ottawa, Canada; 7 Department of Pediatrics, Children’s Hospital of Eastern Ontario, Ottawa, Ontario Canada; 8 Department of Pediatrics, University of Ottawa, Ottawa, Ontario Canada

**Keywords:** The BORN database, The Canadian Institute for Health Information Discharge Abstract Database and National Ambulatory Care Reporting System database, Congenital heart disease, Social-economic status, Maternal deprivation index, Social deprivation index, Rural-urban residence

## Abstract

**Background:**

The risk of congenital heart disease (CHD) has been found to vary by maternal socioeconomic status (SES) and rural-urban residence. In this study, we examined associations of CHD with two maternal SES indicators and stratified the analysis by maternal rural-urban residence.

**Methods:**

This was a population-based retrospective cohort study. We included all singleton stillbirths and live hospital births from April 1, 2012 to March 31, 2018 in Ontario, Canada. We linked the BORN Information System and Canadian Institute for Health Information databases. Multivariable logistic regression models were used to examine associations of CHD with material deprivation index (MDI), social deprivation index (SDI), and maternal residence while adjusting for maternal age at birth, assisted reproductive technology, obesity, pre-pregnancy maternal health conditions, mental health illness before and during pregnancy, substance use during pregnancy, and infant’s sex. MDI and SDI were estimated at a dissemination area level in Ontario and were categorized into quintiles (Q1-Q5).

**Results:**

This cohort study included 798,173 singletons. In maternal urban residence, the p trend (Cochran–Armitage test) was less than 0.0001 for both MDI and SDI; while for rural residence, it was 0.002 and 0.98, respectively. Infants living in the most materially deprived neighbourhoods (MDI Q5) had higher odds of CHD (aOR: 1.21, 95% CI: 1.12–1.29) compared to Q1. Similarly, infants living in the most socially deprived neighbourhoods (SDI Q5) had an 18% increase in the odds of CHD (aOR: 1.18, 95% CI: 1.1–1.26) compared to Q1. Rural infants had a 13% increase in the odds of CHD compared to their urban counterparts. After stratifying by maternal rural-urban residence, we still detected higher odds of CHD with two indices in urban residence but only MDI in rural residence.

**Conclusion:**

Higher material and social deprivation and rural residence were associated with higher odds of CHD. Health interventions and policies should reinforce the need for optimal care for all families, particularly underprivileged families in both rural and urban regions. Future studies should further investigate the effect of social deprivation on the risk of CHD development.

**Supplementary Information:**

The online version contains supplementary material available at 10.1186/s12887-022-03498-6.

## Introduction

Congenital heart disease (CHD) is a major cause of morbidity and mortality among neonates and infants [1, 2]. The prevalence of CHD ranges from 3.7 to 17.5 cases per 1000 births and accounts for 30 to 45% of all congenital anomalies (CAs) globally [1, 3–5]. In Canada, the overall CHD prevalence rate has been estimated to be 12.3 per 1000 total births [6, 7]. In North America, it is estimated that 37% of deaths in infants with CAs are due to CHD [1, 3, 8].

CHD risk has been linked to many factors including genetic, environmental, and behavioural factors [9–11]. Certain social determinants of health have been shown to be important risk factors in determining risk of CHD and a number of other birth outcomes including preterm birth, low birth weight, and small-for-gestational-age and congenital anomalies [12–14]. Specifically, within Canada, recent decades have shown an increase in social inequities, which include significant income and wealth disparities between urban and rural residents [15]. Characterized by limited access to adequate health care and greater exposure to environmental hazards, rural residence has been gaining more attention in recent years due to an increasing number of pregnant people giving birth to infants with CHD [16, 17]. One Canadian study further illustrates this point where the authors showed a link between industrial pollution and place of residence in Alberta, in which occurrences of CHD seemed highest in rural postal codes that were exposed to the highest level of air emissions (RR: 2.6; 95% CI: 1.03—7.0) [18].

In addition, numerous studies have reported that CHD development is associated with maternal socioeconomic status (SES), which was measured by household income, poverty, parental or maternal education level, maternal occupation, and employment status [14, 19–22]. A meta-analysis including data from 33 studies found that lower levels of maternal education and family income, and maternal exposure to certain occupations increased the risk of CHD by 11%, 5% and 51%, respectively [22]. However, the results were not consistent across all geographic areas and SES indicators [22, 23]. The mechanism of this relationship remains unclear. One possible explanation is that mothers with a lower SES level often experience poverty and can only afford to live in an area with disadvantaged environmental living conditions and have less access to healthy foods. In addition, poverty may increase stress levels, which may lead to social drug use and alcohol consumption [14, 20].

Although numerous studies have been published regarding socioeconomic inequities, geographic residence variation, and the risk of CHD, findings are inconsistent [18, 22, 23]. The interrelationship between rural-urban, socioeconomic status (SES), and CHD remains unclear [18, 22, 23]. In the past, we have used multiple SES indicators to measure maternal SES [14]; however, SES has multiple dimensions, and no single SES indicator can represent all perspectives, which may also lead to inconsistent findings regarding associations between SES and CHD [27]. Canadian researchers have developed Canada’s Deprivation Index (CDI), which includes two composite SES indices, maternal deprivation index (MDI) and social deprivation index (SDI), to measure SES at a dissemination area (DA) level [28, 29]. Both MDI and SDI account for six social and economic factors regarding education, employment, income and family structure [29]. MDI focuses on material deprivation such as poverty, while SDI reflects the social isolation of an individual [28]. Canadian researchers have recognized that a composite measurement may be more representative of an individual’s SES rather than a single SES indicator [30–32]. Researchers have demonstrated that CDI can be a proxy of individual level SES measurement and have adopted the indices to estimate SES in public health research in Canada [28, 29, 31–33]. To our knowledge, there are currently no studies that have used these indices (MDI and SDI) to examine the association between SES and CHD. As such, we aimed to fill this gap by measuring the risk of CHD as it associates to SES and urban versus rural status as per the criteria listed in the SDI and MDI.

## Methods

In this study, we aimed to use multiple community SES factors to examine associations of CHD with SES, using a population-based retrospective cohort study with Ontario data from April 1st, 2012 to March 31st, 2018 .

### Study population

The study included late-stage terminations of singleton pregnancies (pregnancies terminated at gestational age ≥ 20 weeks or birthweight ≥500 g) and singleton births (live births and stillbirths) in Ontario hospitals from April 1st, 2012 to March 31st, 2018, with birthweight ≥500 g or gestation ≥20 weeks. Records of  multiple births, and mothers or infants residing outside of Ontario were excluded.

### Data sources

We used the Better Outcomes Registry & Network (BORN), Canadian Institute for Health Information (CIHI)‘s Discharge Abstract Database (DAD), and the CIHI National Ambulatory Care Reporting System (NACRS) databases to conduct this study. BORN collects data on every pregnancy and birth in Ontario through the BORN Information System (BIS) [34–36]. The BORN database captures maternal demographic characteristics and health behaviours; pre-existing maternal health problems; prenatal screening; obstetric complications; intra-partum interventions; fetal anomalies and birth outcomes in pregnancy; labour and birth, and postpartum stages [34, 36]. The data is collected by encounters but also aggregated into maternal pregnancy and infant datasets [34, 35]. Datasets in the BIS were used to perform the analysis including aggregate pregnancy, aggregate infant, antenatal specialty (AS) for high-risk pregnant people clinics, prenatal screen, and prenatal screening follow-up (PSFU) data [14]. BORN continuously strives to ensure high data quality in the BIS through an ongoing data validation process [24, 36], quality checks, and formal training sessions for individuals entering data [35]. BORN data quality analysts monitor the quality including timeliness, reliability, usability, relevance, completeness, and accuracy of BORN data in a dynamic manner [35, 36]. Several papers and reports have been published using these data [34–37].

The DAD and NACRS are maintained by CIHI. The DAD contains inpatients’ demographic, administrative, and clinical information from acute hospital discharge abstracts in Canada, while the NACRS includes health information data from emergency and ambulatory care facilities in Canada [25, 26]. Each year, BORN receives CIHI-DAD and CIHI-NACRS maternal, newborn, and child records (up to 1 year) from acute care and emergency facilities in Ontario [36]. By using these data sources in conjunction with the BIS, we were able to identify infants who had a diagnosis of CHD in hospital up to 1 year of age [14].

### Data linkages and variable definitions

**Fig. 1:**
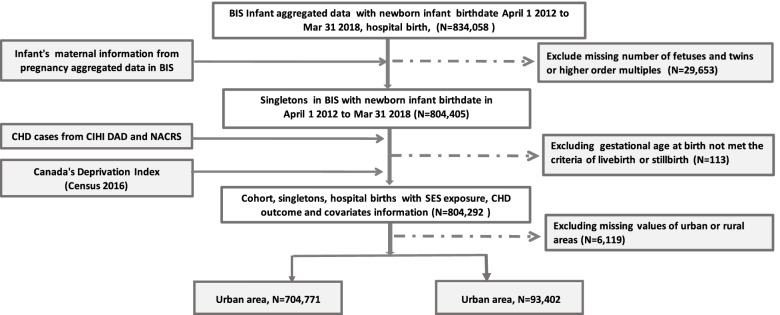
Flowchart of data sources and data linkage for study cohort

The baseline study cohort was obtained from the aggregate infant data of birthdates in the BIS within the required timeframe. This dataset was linked to the aggregate pregnancy data to obtain maternal information including maternal age at delivery, number of fetuses, conception type, pre-pregnancy BMI, pre-pregnancy weight and height, pre-pregnancy maternal health conditions, mental health status before and during pregnancy, social drug intake, and alcohol consumption and smoking status during pregnancy. Multiple birth records (twins, triplets etc.) were excluded from the study cohort. The cohort was further linked to the AS and PSOF to obtain fetal CHD cases. Finally, additional CHD at birth and within 1 year after birth were obtained from the CIHI DAD and NACRS databases with the infant birthdate in the same timeframe [14]. The MDI and SDI indices based on a DA level in Ontario were further linked to the cohort using postal codes of maternal residence. Please see the data linkage flowchart in Fig. 1.

#### Outcome

All prenatally captured CHD cases were identified from AS and PSFU datasets in the BIS. Newborn diagnoses for CHD were collected in the birth child and postpartum child and neonatal intensive care encounters of the BIS and have been aggregated into one infant dataset in the BIS [14]. We also captured the newborn CHD from the aggregate infant dataset. Additional newborn CHD and CHD diagnosed during the first year of infancy were identified from CIHI-DAD and CIHI-NACRS databases [14]. In the BIS, CHD was coded in an anomaly picklist, which was based on clinical diagnosis [14]. In CIHI datasets, CHD was coded using the International Statistical Classification of Diseases and Related Health Problems, 10th Revision, Canadian adaptation (ICD-10-CA) [14]. We included most types of CHD cases and excluded very minor lesions including patent foramen ovale and patent ductus arteriosus. CHD definitions and categorizations in BIS data and CIHI data can be found in the Additional file 1.

#### Covariates

The linked BORN records were used to obtain maternal information including maternal age at delivery, conception type, pre-pregnancy BMI, pre-pregnancy maternal health conditions, mental health illness before or during pregnancy, social drug intake, alcohol consumption and smoking status during pregnancy and infant sex [14, 20]. The covariates were selected based on a literature review and research team members’ clinical experience.

#### Exposures: CDI and maternal rural-urban residence

In this study, we used the CDI - Ontario province (region) to estimate the maternal SES [38]. The CDI dataset was developed and provided by the INSPQ (Public Health Expertise and Reference Centre, Quebec Canada). The latest version was based on the Canada Census 2016 data [38]. CDI has been demonstrated as a valid index to measure SES when there is no individual level SES information in population-based databases [15]. The CDI includes two indices: MDI and SDI, which were calculated using an area-based principal component analysis method and accounting for six SES indicators from the Census data including proportion age 15+ with no high school diploma, employment population ratio of people age 15+, average income of people age 15+, proportion of individuals age 15+ living alone, proportion of individuals age 15+ who are separated, divorced or widowed, and proportion of single-parent families [15, 29]. Both MDI and SDI were calculated using these same six indicators and each indicator was assigned to a different factor weight (a standardized scoring coefficient) [29]. MDI mostly represents material aspects of deprivation including a lower income, lower education level, and lower employment to population ratio, while SDI focuses on the social aspects of deprivation including being separated, divorced, or widowed, and living alone or in a single-parent family [29]. Quintiles of SDI and MDI at a DA level in Ontario were calculated and were treated as composite maternal neighborhood SES factors in two dimensions [29]. DAs are the smallest geographical units available in the 2016 census that are relatively homogeneous to measure SES [38]. CDI data (quintiles of MDI and SDI) were linked to the study cohort using the unique DA identification number.

Maternal rural-urban residence was defined in the 2016 Canadian Census and Postal Code Conversion File Plus (PCCF+) version 7B [39]. We linked maternal postal codes in the BIS to PCCF+ 7B to obtain this variable. This variable was derived using a combination of population size, contiguity, and density [39, 40].

### Statistical analysis

Distributions of exposure variables and covariates by CHD was described first. The p trend Cochran-Armitage test was used to assess the trend of association between CDI and CHD. Multivariable logistic regression models were performed to examine the relationships between the MDI, SDI, rural-urban residence, and CHD. SDI and MDI were estimated at a DA level and categorized by quintiles. Adjustments were made for maternal age at birth, assisted reproductive technology, obesity, pre-existing maternal health conditions, mental health illness before and during pregnancy, substance use during pregnancy, and infant’s sex. We did not rely on a statistical significance to keep a variable in the multivariable logistic regression models. As such, all covariables were forced in the analyses [41]. The MDI, SDI and rural-urban residence were in one multivariable model to estimate the ORs and 95% confidence intervals (CI) while adjusting for covariates. Interactions between rural-urban residence with MDI and SDI on CHD were evaluated in a multivariable regression model. The relationships between MDI and SDI, and CHD were further assessed by stratification of the maternal rural-urban residence. All data linkages and analyses were performed using SAS 9.4 (SAS Institute Inc., Cary NC).

## Results

The study cohort retrieved from the BIS infant aggregated data consisted of 834,058 newborn infants born between April 1, 2012 and March 31, 2018 (Fig. 1). Data were subsequently linked with the infant’s maternal information, which led to 29,653 being excluded due to missing number of fetuses and twins or higher order multiples. A resulting 804,405 singletons from the BIS went on to be evaluated for SES exposure, CHD outcome and covariate information, which led to 113 being excluded due to not meeting the criteria of live- or stillbirth. Another 6119 were excluded due to missing values for rural-urban areas. A total of 704,771 infants were classified as livin g in an urban area and 93,402 in a rural area. In total, 9649 CHD cases (1.21% of 798,173) were identified in this cohort. 

Table 1 shows the maternal and infant characteristics of the study population. Individuals in urban residences were found to have a lower maternal pre-pregnancy BMI of 24.96 kg/m^2^ compared to 25.82 kg/m^2^ for mothers in rural areas. Mothers in urban residences were also found to be older (mean of 30.77 years of age, with 38.93% under 30 years) compared to a mean of 29.07 years of age (with 52.72% under 30 years) for those living in rural residence. Likewise, pregnant people living in an urban residence had lower prevalence rates of obesity (14.72% versus 19.20%), smoking / drug use / alcohol consumption (10.95% versus 19.61%), a mental health illness in pre-pregnancy or during pregnancy (14.15% versus 20.21%), and a pre-pregnancy maternal health conditions (18.38% versus 20.28%) when compared to pregnant people living in rural residence. By contrast, they had higher prevalence rates of ART conception (3.25%) com pared to 2.54% of mothers in rural residences. There was no significant difference between rural and urban in the number of male and female babies observed.

**Table 1:** Maternal and infant characteristics of study population

	Urban residence		Rural residence		*P* value
	N	%	N	%	
Maternal pre-pregnancy body mass index (BMI) in kg/m^2^, mean ± SD	24.96	6.11	25.82	6.52	< 0.0001
Maternal age at birth in years, mean ± SD	30.77	5.28	29.07	5.38	< 0.0001
< 30	274,378	38.93	49,241	52.72	< 0.0001
30–34	260,082	36.90	29,610	31.70	
35 and over	169,809	24.09	14,529	15.56	
Obesity, BMI ≥ 30 kg/m^2^, yes, # (%)					
Yes	103,747	14.72	17,929	19.20	<.0001
No	512,951	72.78	66,311	71.00	
Missing	88,073	12.50	9162	9.81	
ART derived pregnancy					<.0001
No	681,891	96.75	91,025	97.46	
Yes	22,880	3.25	2377	2.54	
Maternal smoking or social drug use or alcohol consumption during pregnancy					<.0001
Yes	77,164	10.95	18,313	19.61	
No	608,983	86.41	74,565	79.83	
Missing	18,624	2.64	524	0.56	
All types of mental health illness in pre-pregnancy or during pregnancy					<.0001
Yes	99,735	14.15	18,874	20.21	
No	605,036	85.85	74,528	79.79	
Pre-pregnancy health conditions , yes, # (%)					<.0001
Yes	129,571	18.38	18,946	20.28	
No	575,200	81.62	74,456	79.72	
Infant sex					0.35
Male	361,547	51.30	48,053	51.45	
Female	342,519	48.60	45,225	48.42	

Table 2 illustrates the associations between social and material deprivation indices and rural or urban residence by crude and adjusted ORs. The odds of CHD for infants was highest in the fifth quintile, where the mothers lived in the most deprived neighbourhoods, with an adjusted OR of 1.21 (CI 95%; 1.12–1.29) compared to infants whose mothers lived in the first quintile (least deprived) neighbourhoods on the MDI. Similarly, the SDI showed a comparable result with infants from the most deprived (Q5) neighbourhoods at the highest odds of CHD (aOR 1.18; CI 95% 1.1–1.26) compared to their counterparts in the least deprived (Q1) neighbourhoods. Other variables that were associated with CHD included rural residence (aOR 1.13; CI 95% 1.06–1.21), maternal age of 35 years or older (aOR 1.27; CI 95% 1.2–1.34) compared to pregnant people 30 years and younger, obesity (aOR 1.27; CI 95% 1.2–1.34), use of ART (aOR 1.13; CI 95% 1.01–1.26), smoking/alcohol/drug use during pregnancy (aOR 1.31; CI 95% 1.23–1.39), presence of a mental health illness before or during pregnancy (aOR 1.23; CI 95% 1.17–1.3), presence of a pre-pregnancy maternal health condition (aOR 1.73; CI 95% 1.65–1.82), and male infant sex (aOR 1.1; CI 95% 1.06–1.15). The p trend (Cochran-Armitage test) was < 0.0001 for both MDI and SDI. The interaction tests between rural-urban residence with MDI and SDI were not statistically significant. The *p* values of the interaction test between rural-urban residence and MDI and between rural-urban residence and SDI were 0.11 and 0.34, respectively.

**Table 2:** Associations between deprivation indices and congenital heart disease

Variable	Crude OR	Adjust OR
Material deprivation index		
1 (lowest)	Reference	Reference
2	1.04 (0.96–1.12)	1.05 (0.97–1.13)
3	1.11 (1.03–1.19)	1.12 (1.04–1.20)
4	1.13 (1.05–1.21)	1.15 (1.07–1.23)
5 (highest)	1.20 (1.12–1.28)	1.21 (1.12–1.29)
Social deprivation index^a^		
1 (lowest)	Reference	Reference
2	1.10 (1.02–1.18)	1.08 (1–1.15)
3	1.07 (1–1.15)	1.04 (0.97–1.12)
4	1.14 (1.06–1.22)	1.09 (1.02–1.17)
5 (highest)	1.25 (1.17–1.34)	1.18 (1.1–1.26)
Rural residence		
Yes	1.12 (1.06–1.2)	1.13 (1.06–1.21)
No	Reference	Reference
Maternal age at birth		
< 30 years	Reference	Reference
30–34 years	0.94 (0.90–0.99)	1.00 (0.95–1.05)
> 35 years	1.22 (1.16–1.28)	1.27 (1.20–1.34)
Obesity		
Yes	1.41 (1.33–1.48)	1.27 (1.20–1.34)
No	Reference	Reference
Unknown	1.1 (1.03–1.18)	1.13 (1.06–1.21)
ART derived pregnancy		
Yes	1.25 (1.12–1.39)	1.13 (1.01–1.26)
No	Reference	Reference
Smoking, alcohol, or drug use during pregnancy		
Yes	1.46 (1.38–1.54)	1.31 (1.23–1.39)
No	Reference	Reference
All types of mental health illness in pre-pregnancy or during pregnancy		
Yes	1.45 (1.37–1.53)	1.23 (1.17–1.30)
No	Reference	Reference
Pre-pregnancy maternal health conditions		
Yes	1.86 (1.77–1.94)	1.73 (1.65–1.82)
No	Reference	Reference
Infant sex		
Male	1.10 (1.06–1.15)	1.10 (1.06–1.15)
Female	Reference	Reference

^a^ p trend (Cochran–Armitage test) < 0.0001

Table 3 shows the associations between social and material deprivation indices by rural or urban residence. The odds of CHD for infants was highest in the most deprived neighbourhoods for mothers living in both urban (aOR 1.20; CI 95% 1.11–1.29) and rural (aOR 1.30; CI 95% 1.06–1.61) residences compared to their more privileged counterparts when the material deprivation index was used. In contrast, the SDI showed different results where the odds of CHD was significant in the most deprived neighbourhoods for mothers in urban residences (aOR 1.20; CI 95% 1.11–1.29), but not those living in rural residences (aOR 0.68; CI 95% 0.37–1.24) compared to the least deprived neighbourhoods.

**Table 3:** Associations between deprivation indices and congenital heart disease by rural or urban residence

Variable	Urban residence	Rural residence
	Adjusted OR	Adjusted OR
Material deprivation index^a^		
1 (lowest)	Reference	Reference
2	1.07 (0.99–1.16)	0.93 (0.77–1.13)
3	1.11 (1.03–1.2)	1.17 (0.97–1.41)
4	1.16 (1.07–1.25)	1.04 (0.84–1.28)
5 (highest)	1.20 (1.11–1.29)	1.30 (1.06–1.61)
Social deprivation index^b^		
1 (lowest)	Reference	Reference
2	1.09 (1.00–1.18)	1.01 (0.86–1.19)
3	1.06 (0.98–1.15)	0.96 (0.81–1.14)
4	1.11 (1.03–1.19)	1.00 (0.80–1.25)
5 (highest)	1.20 (1.11–1.29)	0.68 (0.37–1.24)
Maternal age at birth		
< 30 years	Reference	Reference
30–34 years	0.99 (0.94–1.05)	1.05 (0.91–1.2)
> 35 years	1.25 (1.18–1.33)	1.4 (1.20–1.64)
Obesity		
Yes	1.29 (1.22–1.37)	1.15 (0.99–1.32)
No	Reference	Reference
Unknown	1.13 (1.06–1.22)	1.10 (0.90–1.34)
ART derived pregnancy		
Yes	1.16 (1.03–1.30)	0.90 (0.62–1.32)
No	Reference	Reference
Smoking, alcohol or drug use during pregnancy		
Yes	1.31 (1.23–1.4)	1.25 (1.08–1.44)
No	Reference	Reference
All types of mental health illness in pre-pregnancy or during pregnancy		
Yes	1.22 (1.15–1.30)	1.26 (1.10–1.45)
No	Reference	Reference
Pre-pregnancy maternal health conditions		
Yes	1.74 (1.66–1.83)	1.68 (1.48–1.92)
No	Reference	Reference
Infant sex		
Male	1.09 (1.04–1.14)	1.21 (1.07–1.36)
Female	Reference	Reference

^a^ p trend (Cochran–Armitage test) is 0.002 for rural area and < 0.0001 for urban area

^b^ p trend (Cochran–Armitage test) is 0.98 for rural area and < 0.0001 for urban area

Other variables associated with CHD for infants with mothers living in urban residences include maternal age of 35 years or older (aOR 1.25; CI 95% 1.18–1.33), obesity (aOR 1.29; CI 95% 1.22–1.37), ART derived pregnancy (aOR 1.16; CI 95% 1.03–1.3), smoking/alcohol/drug use during pregnancy (aOR 1.31; CI 95% 1.23–1.4), mental health illness (aOR 1.22; CI 95% 1.15–1.3), pre-pregnancy maternal health conditions (aOR 1.74; CI 95% 1.66–1.83), and male infant sex (aOR 1.09; CI 95% 1.04–1.14).

Similarly, for mothers living in rural residences, maternal age of 35 years or older (aOR 1.40; CI 95% 1.2–1.64), smoking/alcohol or drug use during pregnancy (aOR 1.25; CI 95% 1.08–1.44), mental health illness (aOR 1.26; CI 95% 1.1–1.45), pre-pregnancy health conditions (aOR 1.68; CI 95% 1.48–1.92), and male infant sex (aOR 1.21; CI 95% 1.07–1.36) were also found to be associated with CHD in infants. However, obesity (aOR 1.15; CI 95% 0.99–1.32) and ART derived pregnancy (aOR 0.90; CI 95% 0.62–1.32) were not found to be associated with CHD in infants with mothers in rural residences. The p-trend (Cochran-Armitage test) was 0.002 for rural area and < 0.0001 for urban area when considering MDI alone. However, when SDI was used instead, the p-trend (Cochran-Armitage test) turned out to be 0.98 for rural area and < 0.0001 for urban area.

Table 4 shows the prevalence of congenital heart disease by material deprivation index and s ocial deprivation index and maternal residence. The highest rates of CHD were found when using the MDI in the most deprived rural neighbourhoods at 1.62%, compared to only 1.29% for the most deprived urban neighbourhoods. In contrast, when using the SDI, the most deprived urban neighbourhoods had a prevalence rate of 1.34%, compared to 1.05% for the most deprived rural residences. Of note is the smaller number of infants found with CHD in the rural population compared to the urban population. Overall, however, both urban and rural infants experienced a similar trend of increasing CHD with increasing neighbourhood deprivation, with the only exception being found in SDI’s most deprived rural neighbourhood where only 11 cases of CHD were found for a prevalence rate of 1.05%. Prevalence rates for Q1 to Q4 for SDI rural residence were found to be higher than the ones observed in the urban sample.

**Table 4:** Prevalence of congenital heart disease by material deprivation index and social deprivation index and maternal residence

Variable	Urban residence			Rural residence		
	Total number	Number of CHD	%	Total number	Number of CHD	%
Material deprivation index						
Q1 (lowest)	113,227	1195	1.06	14,708	183	1.24
Q2	120,209	1373	1.14	22,347	255	1.14
Q3	129,567	1527	1.18	22,354	327	1.46
Q4	142,087	1757	1.24	14,986	189	1.26
Q5 (highest)	170,406	2198	1.29	13,494	218	1.62
Missing	29,275		4.33	5513		6.27
Social deprivation index						
Q1 (lowest)	127,768	1349	1.06	20,921	276	1.32
Q2	109,178	1245	1.14	32,581	441	1.35
Q3	125,233	1442	1.15	24,865	324	1.30
Q4	152,558	1863	1.22	8475	120	1.42
Q5 (highest)	160,759	2151	1.34	1047	11	1.05
Missing	29,275		4.33	5513		6.27

*CHD* Congenital heart disease

## Discussion

In this study, we found that both MDI and SDI were associated with CHD. After adjusting for covariates of maternal age at birth, assisted reproductive technology, obesity, pre-existing maternal health conditions, mental health illness before and during pregnancy, substance use during pregnancy, rural or urban residence, and infant’s sex, we found that both higher material and social deprivation indices and maternal rural residence likely increased the odds of CHD. When the cohort was stratified by maternal rural and urban residence, we still found that MDI and SDI in urban residence and MDI in rural residence were associated with higher odds of CHD; however, the data did not sufficiently support that SDI was associated with CHD in maternal rural residence. In maternal urban residential areas, both material and social deprivation indices showed a significant linear trend (“dose response”) of association with CHD. In maternal rural residential areas, only the MDI showed a linear relationship with CHD.

SDI and MDI are two composite in dices that are representative of SES in two perspectives [29, 42, 43]. The material disadvantages (or deprivation) mainly reflect lower education, poverty, and a lower employment rate, which suggests financial disadvantages, whereas social disadvantages (or deprivation) reflect marital separation, divorced or widowed marital status, living alone or living in a single-parent family. These social disadvantages imply social fragmentation and social isolation [29, 44]. Many studies have discussed the potential mechanism that people who lack financial resources tend to reside in areas with disadvantaged living environments, consume low quality food, and have higher stress levels, which could increase the risk of CHD [3, 6, 11, 14, 15, 19, 21]. Although very few studies have been done on the association between social deprivation and CHD, previous studies have reported that social support during pregnancy could reduce stress and anxiety, which could potentially decrease the risk of adverse birth outcomes [45]. As such, these indices help to delineate which aspects of SES may influence the development of CHD and help to propose potential mechanisms that may be underlying its etiology.

Overall, the findings of this study where MDI and SDI were used are consistent with other studies that used different SES indicators [14, 46]. In our previously published study, we found that infants whose mothers lived in the lowest income neighbourhoods had a higher likelihood of developing CHD (adjusted OR: 1.29, 95% CI: 1.20–1.38) when compared to infants with mothers who lived in a more affluent neighbourhood. Similarly, infants whose mothers lived in the neighbourhoods with the lowest percentage of people with a university or higher degree had a higher chance of CHD (adjusted OR: 1.34, 95% CI: 1.24–1.44) when compared to those living in the neighbourhoods with the highest percentage of people with a university of higher degree [14]. Studies from other countries showed similar results [22, 23]. One recent study conducted in the United States found the incidence of CHD among infants to be higher in the most socially and economically disadvantaged neighbourhoods (quartile 4) compared to the least disadvantaged neighbourhoods (quartile 1) (adjusted OR 1.31, 95% CI: 1.21–1.41) [46].

Our study also suggests that living in rural residences might slightly increase the odds of CHD. The mechanism is unclear since there is lack of research regarding the association between rural-urban residency and CHD. Nevertheless, previous research has shown that rural isolation and limited access to healthcare services may be a potential reason behind rural-urban maternal health inequities [13, 47–49]. One study in the Canadian province of British Columbia looked at various perinatal outcomes among expecting mothers in both rural and urban areas and found that rural areas have higher rates of severe maternal morbidity (aOR 1.15, 95% CI; 1.03–1.28) and severe neonatal morbidity (aOR 1.14, 95% CI; 1.02–1.29) [50]. Similarly, a U.S. study reported a 9% greater probability of severe maternal morbidity and mortality among rural residents when compared to urban residents  [51]. More studies are needed to explore the mechanism in the future.

There are inconsistent findings regarding inequities between rural and urban residence, with varying dimensions of SES being noted in the literature [32, 52, 53]. Our study showed that the association between the MDI and CHD is similar between urban and rural residence, while no association was detected between SDI and CHD in rural area (Table 3). The mechanism is unknown. Compared to social deprivation or family isolation, unavailability of health care resources or distance from a tertiary maternal and fetal care center in rural areas might be a more dominant risk factor for CHD in rural residences. However, the ORs regarding association between MDI and CHD presented for rural residence had much wider confidence intervals compared to their urban counterparts, which may indicate less certainty with these results due to a much smaller sample size in rural areas (Tables 3 and 4), especially considering there were only 11 CHD cases in the Q5 category of SDI. As such, the lack of precision makes it difficult to ascertain whether there are significant differences between rural or urban residences.

This study has several strengths. We included all data from singleton births from the 2012–2013 fiscal year to the 2017–2018 fiscal year in Ontario, Canada, which helped provide a larger sample to improve the precision of the results. As well, the CHD cases were determined by linking multiple data sources including those identified in prenatal, postnatal, or birth records and those identified up to 1 year of infancy to obtain more complete information. Moreover, our stratification of the data by rural and urban residence helped to further elucidate the effect of geography on covariate associations with CHD, which, to our knowledge, has been lacking in the literature. Furthermore, an individual’s SES is complex with multiple dimensions, and a single SES indicator may not reflect a person’s SES in multiple dimensions. As such, a strength of our study was the use of two composite indices, MDI and SDI.

Nevertheless, there were some limitations that need to be considered. Both MDI and SDI were estimated at a small area (DA) level. As such, there was potential misclassification of SES measurements. However, DAs are the smallest geographic unit with around 400–700 people per DA in Canada [14]. MDI and SDI measurement at a DA level have been considered as good proxies when individual level SES information is not available [29]. In addition, literature shows that environmental hazards and unhealthy diet may also increase the risk of CHD [1, 3]. However, due to data limitations, we were not able to account for these potential confounders in the multivariable regression analysis. Another important note is that the data obtained from the birth registry and health administrative data are not used solely for specific research projects, and so, there is a potential that some CHD outcomes were misclassified. However, it is likely non-differential, which would decrease the effect size observed. Furthermore, the outcome group CHDs might represent an etiologically heterogeneous collection of various CHD related phenotypes and genotypes [54]. However, due to a small sample size and data limitations, we were not able to conduct a study to examine associations between SES and certain types of CHD. In our future studies, when there is sufficient data and sample size, we will consider developing a study to examine relationships between CHD subtypes and SES. Moreover, we might have missed the CHD cases that were diagnosed after 1 year of age [55]. For example, some ventricular septal defects and atrial septal defect cases or mild lesions can present later in life. As such, we may underreport the CHD cases and prevalence rate [55]. Finally, this study did not include terminations before 20 weeks gestation due to BORN data limitations, which decreased the reported prevalence rate of CHD in this study. In the future, after we capture more complete data, we will include those early termination records to examine the association between SES and the risk of CHD, and to compare CHD prevalence rates and early terminations in the rural-urban environment. This may help to explain to some extent why the CHD rates are higher in rural residences.

## Conclusions

In summary, we found that both MDI and SDI showed a trend of association with CHD. After adjusting for covariates such as maternal age at birth, assisted reproductive technology, obesity, pre-existing maternal health conditions, mental health illness before and during pregnancy, rural or urban residence, and infant’s sex, we found that both higher material and social deprivation indices and maternal rural residence likely increased the odds of CHD. When the cohort was stratified by maternal rural and urban residence, we still found that MDI and SDI in urban residence and MDI in rural residence were associated with higher odds of CHD; however, the data did not sufficiently support that SDI was associated with CHD in maternal rural residence. Health interventions and policies should reinforce the need for optimal care for all families, particularly underprivileged families in both rural and urban regions. It might be difficult to change an individual’s SDI (family isolation); however, public health care decision makers could instead aim to change an individual’s MDI to minimize SES gaps for CHD interventions. Future studies should investigate further the effect of social deprivation on the risk of CHD development.

## Supplementary Information

**Additional file 1.**

## Data Availability

The data analyzed during this study is held securely at the prescribed registry BORN Ontario. Data sharing regulations prevent this data from being made available publicly due to the personal health information in the datasets. Enquiries regarding BORN data must be directed to BORN Ontario (Science@BORNOntario.ca).

## Abbreviations

CHD: Congenital Heart Disease
SES: Socioeconomic Status
BORN: Better Outcomes Registry & Network
CIHI: Canadian Institute for Health Information
ART: Assisted Reproductive Technology
BIS: BORN Information System
BMI: Body Mass Index
OR: Odds Ratio
aOR: Adjusted Odds Ratio
CA: Congenital Anomalies
RR: Relative Risk
AS: Antenatal Speciality
PSFU: Prenatal Screening Follow-Up
DAD: Discharge Abstract Database
NACRS: National Ambulatory Care Reporting System
PCCF+: Postal Code Conversion File Plus
ICD-10-CA: International Statistical Classification of Diseases and Related Health Problems, 10th Revision, Canada Adaptation
CDI: Canada’s Deprivation Index
SDI: Social Deprivation Index
MDI: Material Deprivation Index
DA: Dissemination Area
REB: Research Ethics Board
WHO: World Health Organization

## References

[1] Hoffman JIE (2013). The global burden of congenital heart disease : review article. Cardiovasc J Afr.

[2] Kochanek KD, Tejada-Vera B. Deaths: Final Data for 2014. 122. 27378572

[3] Sayasathid J, Sukonpan K, Somboonna N. Epidemiology and etiology of congenital heart diseases. Congenital Heart Disease - Selected Aspects Epub ahead of print January 18, 2012. DOI: 10.5772/27083.

[4] Irvine B, Luo W, León JA (2015). Congenital anomalies in Canada 2013: a perinatal health surveillance report by the Public Health Agency of Canada’s Canadian perinatal surveillance system. Health Promot Chronic Dis Prev Can.

[5] Mulder BJM (2012). Epidemiology of adult congenital heart disease: demographic variations worldwide. Neth Heart J.

[6] Parnell AS, Correa A (2017). Analyses of trends in prevalence of congenital heart defects and folic acid supplementation. J Thorac Dis.

[7] Liu Shiliang, Joseph K.S., Luo Wei, León Juan Andrés, Lisonkova Sarka, Van den Hof Michiel, Evans Jane, Lim Ken, Little Julian, Sauve Reg, Kramer Michael S. (2016). Effect of Folic Acid Food Fortification in Canada on Congenital Heart Disease Subtypes. Circulation.

[8] Botto Lorenzo D, Correa Adolfo (2003). Decreasing the burden of congenital heart anomalies: an epidemiologic evaluation of risk factors and survival. Progress in Pediatric Cardiology.

[9] Diogenes TCP, Mourato FA, de Lima Filho JL (2017). Gender differences in the prevalence of congenital heart disease in Down’s syndrome: a brief meta-analysis. BMC Med Genet.

[10] van der Linde D, Konings EEM, Slager MA, et al. Birth prevalence of congenital heart disease worldwide. Epub ahead of print 2011. DOI: 10.1016/j.jacc.2011.08.025. 10.1016/j.jacc.2011.08.025 22078432

[11] Lage K, Greenway SC, Rosenfeld JA (2012). Genetic and environmental risk factors in congenital heart disease functionally converge in protein networks driving heart development. Proc Natl Acad Sci.

[12] Auger N, Authier M-A, Martinez J (2009). The association between rural-urban continuum, maternal education and adverse birth outcomes in Québec, Canada. J Rural Health.

[13] Luo Z-C, Wilkins R (2008). Degree of rural isolation and birth outcomes. Paediatr Perinat Epidemiol.

[14] Miao Q, Dunn S, Wen SW (2021). Neighbourhood maternal socioeconomic status indicators and risk of congenital heart disease. BMC Pregnancy Childbirth.

[15] Chan E, Serrano J, Chen L (2015). Development of a Canadian socioeconomic status index for the study of health outcomes related to environmental pollution. BMC Public Health.

[16] Pinto NM, Keenan HT, Minich LL (2012). Barriers to prenatal detection of congenital heart disease: a population-based study. Ultrasound Obstet Gynecol.

[17] McKenzie LM, Guo R, Witter RZ (2014). Birth outcomes and maternal residential proximity to natural gas development in rural Colorado. Environ Health Perspect.

[18] Ngwezi DP, Hornberger LK, Serrano-Lomelin J (2018). Industrial developmental toxicants and congenital heart disease in urban and rural Alberta, Canada. Challenges.

[19] Li X, Sundquist J, Hamano T (2016). Neighbourhood deprivation, individual-level and familial-level socio-demographic factors and risk of congenital heart disease: a Nationwide study from Sweden. Int J Behav Med.

[20] Miao Q, Dunn S, Wen SW, et al. Association of maternal socioeconomic status and race with risk of congenital heart disease: a population-based retrospective cohort study in Ontario, Canada. BMJ Open; 12. Epub ahead of print February 1, 2022. DOI: 10.1136/BMJOPEN-2021-051020. 10.1136/bmjopen-2021-051020 PMC8808396 35105571

[21] Deguen S, Kihal W, Jeanjean M (2016). Neighborhood deprivation and risk of congenital heart defects, neural tube defects and orofacial clefts: a systematic review and Meta-analysis. PLoS One.

[22] Yu D, Feng Y, Yang L (2014). Maternal socioeconomic status and the risk of congenital heart defects in offspring: a Meta-analysis of 33 studies. PLoS One.

[23] Ngwezi D, Hornberger L, Vargas O, Alvaro. The role of socioeconomic status and the development of congenital heart disease: a scoping review. Advanc Pediatr Res. 2018;5. 10.24105/apr.2018.5.19.

[24] Dunn S, Sprague AE, Grimshaw JM, et al. A mixed methods evaluation of the maternal-newborn dashboard in Ontario: Dashboard attributes, contextual factors, and facilitators and barriers to use: A study protocol. Implement Sci; 11. Epub ahead of print 2016. DOI: 10.1186/s13012-016-0427-1. 10.1186/s13012-016-0427-1 PMC4855363 27142655

[25] Discharge Abstract Database metadata (DAD) | CIHI, https://www.cihi.ca/en/discharge-abstract-database-metadata-dad (accessed November 29, 2021).

[26] National Ambulatory Care Reporting System metadata (NACRS) | CIHI, https://www.cihi.ca/en/national-ambulatory-care-reporting-system-metadata-nacrs (accessed November 29, 2021).

[27] Braveman PA, Cubbin C, Egerter S (2005). Socioeconomic status in Health Research: one size does not fit all. JAMA.

[28] Auger N, Park AL, Gamache P (2012). Weighing the contributions of material and social area deprivation to preterm birth. Soc Sci Med.

[29] Pampalon R, Hamel D, Gamache P, et al. An area-based material and social deprivation index for public health in Québec and Canada. *Canadian journal of public health = Revue canadienne de sante publique*; 103. Epub ahead of print 2012. DOI: 10.1007/BF03403824. 10.1007/BF03403824 PMC6973787 23618066

[30] Palayew A, Schmidt AM, Saeed S (2021). Estimating an individual-level deprivation index for HIV/HCV coinfected persons in Canada. PLoS One.

[31] Hwang J, Rudnisky C, Bowen S (2017). Measuring socioeconomic inequalities in eye care services among patients with diabetes in Alberta, Canada, 1995-2009. Diabetes Res Clin Pract.

[32] Pampalon R, Hamel D, Gamache P (2010). Health inequalities in urban and rural Canada: comparing inequalities in survival according to an individual and area-based deprivation index. Health Place.

[33] Amjad S, Chandra S, Osornio-Vargas A (2019). Maternal area of residence, socioeconomic status, and risk of adverse maternal and birth outcomes in adolescent mothers. J Obstet Gynaecol Can.

[34] Sprague AE, Sidney D, Darling EK (2018). Outcomes for the first year of Ontario’s birth center demonstration project. J Midwife Women’s Health.

[35] Dunn S, Lanes A, Sprague AE (2019). Data accuracy in the Ontario birth Registry: a chart re-abstraction study. BMC Health Serv Res.

[36] Miao Q, Fell DB, Dunn S (2019). Agreement assessment of key maternal and newborn data elements between birth registry and clinical administrative hospital databases in Ontario, Canada. Arch Gynecol Obstet.

[37] Dunn S, Sprague AE, Grimshaw JM, et al. A mixed methods evaluation of the maternal-newborn dashboard in Ontario: Dashboard attributes, contextual factors, and facilitators and barriers to use: A study protocol. Implementation Science; 11. Epub ahead of print 2016. DOI: 10.1186/s13012-016-0427-1. 10.1186/s13012-016-0427-1 PMC4855363 27142655

[38] Une production du Material and social deprivation index: A summary OVERVIEW OF THE METHODOLOGY, http://www.inspq.qc.ca. (accessed October 24, 2021).

[39] Statistics Canada. Postal CodeOM conversion file plus (PCCF+) version 7B, Reference Guide. Statistics Canada.

[40] Statistics Canada. Census of Population Reference Product, www.statcan.gc.ca (2016, accessed October 24, 2021).

[41] Bursac Z, Gauss CH, Williams DK (2008). Purposeful selection of variables in logistic regression. Source Code Biol Med.

[42] Matheson FI, Dunn JR, Smith KLW, et al. Development of the Canadian Marginalization Index: A New Tool for the Study of Inequality. 103: 12–16. 10.1007/BF03403823 PMC6973681 23618065

[43] Pampalon R, Hamel D, Gamache P, Raymond G (2009). A deprivation index for health planning in Canada. Chronic Dis Can.

[44] Hamel D, Pampalon R. A deprivation index for health planning in Canada Obesity-Spatial and socioeconomic analyses View project Terrain parks project View project. Chronic Diseases in Canada; 29. Epub ahead of print 2009. DOI: 10.24095/hpcdp.29.4.05. 19804682

[45] Hetherington E, Doktorchik C, Premji SS (2015). Preterm birth and social support during pregnancy: a systematic review and Meta-analysis. Paediatr Perinat Epidemiol.

[46] Peyvandi S, Baer RJ, Chambers CD, et al. Environmental and Socioeconomic Factors Influence the Live-Born Incidence of Congenital Heart Disease: A Population-Based Study in California. J Am Heart Assoc; 9. Epub ahead of print April 21, 2020. DOI: 10.1161/JAHA.119.015255. 10.1161/JAHA.119.015255 PMC7428546 32306820

[47] Brundisini F, Giacomini M, DeJean D (2013). Chronic disease patients’ experiences with accessing health Care in Rural and Remote Areas: a systematic review and qualitative Meta-synthesis. Ontario Health Technol Assess Ser.

[48] Say L, Raine R. A systematic review of inequalities in the use of maternal health care in developing countries: examining the scale of the problem and the importance of context Public health reviews. Bull World Health Organ; 85. Epub ahead of print 2007. DOI: 10.2471/BLT.06.035659. 10.2471/BLT.06.035659 PMC2636485 18038064

[49] Stoll K, Kornelsen J (2014). Midwifery Care in Rural and Remote British Columbia: a retrospective cohort study of perinatal outcomes of rural parturient women with a midwife involved in their care, 2003 to 2008. J Midwife Women’s Health.

[50] Lisonkova S, Haslam MD, Dahlgren L (2016). Maternal morbidity and perinatal outcomes among women in rural versus urban areas. CMAJ.

[51] Kozhimannil KB, Interrante JD, Henning-Smith C, et al. Rural-Urban Differences In Severe Maternal Morbidity And Mortality In The US, 2007–15. https://doi.org/101377/hlthaff201900805 2019; 38: 2077–2085. 10.1377/hlthaff.2019.00805 31794322

[52] Gilthorpe MS, Wilson RC (2003). Rural/urban differences in the association between deprivation and healthcare utilisation. Soc Sci Med.

[53] Gartner A, Farewell D, Roach P (2011). Rural/urban mortality differences in England and Wales and the effect of deprivation adjustment. Soc Sci Med.

[54] Digilio MC, Marino B (2016). What is new in genetics of congenital heart defects?. Front Pediatr.

[55] Hoffman JIE, Kaplan S (2002). The incidence of congenital heart disease. J Am Coll Cardiol.

