# Supplementary material for: Associations of congenital heart disease with deprivation index by rural-urban maternal residence: a population-based retrospective cohort study in Ontario, Canada
Source: BMC Pediatr. 2022 Aug 5;22:476. doi: 10.1186/s12887-022-03498-6 (PMC9356510; doi:10.1186/s12887-022-03498-6)
Supplement: Supplementary file 1 — Additional file 1. [file 12887_2022_3498_MOESM1_ESM.docx]

**Additional file 1. Congenital heart disease coding using ICD-10-CA**

| Including Q20 toQ23 | |  |  | |  |  |  |
| --- | --- | --- | --- | --- | --- | --- | --- |
| Q20 | Congenital malformations of cardiac chambers and connections | | | | | | |
| Q21 | Congenital malformations of cardiac septa | | | | |  |  |
| Q22 | Congenital malformations of pulmonary and tricuspid valves | | | | | |  |
| Q23 | Congenital malformations of aortic and mitral valves | | | | | |  |
|  |  | | | |  |  |  |
| Q24 | Other congenital malformations of heart | | | |  |  |  |
| Including Q24.0, Q24.8, Q24.9, Q24.2 to Q24.5 | | | | | |  |  |
|  | | | | |  |  |  |
| Q25 Congenital malformations of great arteries Including Q25.1 to Q25.9 | | | | |  |  |  |
|  | | | | |  |  |  |
| Q26 Congenital malformations of great veins | | | | |  |  |  |
| Include Q26.0 to Q26.4 | | | |  |  |  |  |
